# Anti-phospholipid antibodies nephropathy is associated with an increased risk of kidney failure: a systematic literature review and meta-analysis

**DOI:** 10.1093/ckj/sfae302

**Published:** 2024-10-07

**Authors:** Ariela Hoxha, Marco Lovisotto, Nicola Perin, Federico Nalesso, Dorella Del Prete, Paolo Simioni

**Affiliations:** Internal Medicine Unit, Thrombotic and Hemorrhagic Center, Department of Medicine, University of Padua, Padua, Italy; Internal Medicine Unit, Thrombotic and Hemorrhagic Center, Department of Medicine, University of Padua, Padua, Italy; Internal Medicine Unit, Thrombotic and Hemorrhagic Center, Department of Medicine, University of Padua, Padua, Italy; Nephrology Unit, Department of Medicine, University of Padua, Padua, Italy; Nephrology Unit, Department of Medicine, University of Padua, Padua, Italy; Internal Medicine Unit, Thrombotic and Hemorrhagic Center, Department of Medicine, University of Padua, Padua, Italy

**Keywords:** anti-phospholipid antibodies, anti-phospholipid antibodies nephropathy, anti-phospholipid syndrome, lupus anticoagulant, thrombotic microangiopathy

## Abstract

**Background:**

Anti-phospholipid antibodies nephropathy (aPL-N) is a complex feature of anti-phospholipid syndrome due to microvascular lesions. Renal prognosis and predictors of outcome are not yet known.

**Methods:**

We performed a systematic review of the literature (February 2006–January 2024) using the PubMed, Scopus, Cochrane Library and EMBASE databases. Two reviewers independently conducted literature screening and data extraction in a blinded, standardized manner. A random effects model was used to pool odds ratios (ORs) [with 95% confidence interval (CI)] for the primary analysis, the risk of kidney failure. Subgroup analyses were performed for clinical and laboratory features that predicted renal outcomes. Heterogeneity was assessed by I^2^.

**Results:**

Six records involving 709 patients were included in the meta-analysis. Biopsy-proven aPL-N was found in 238/832 (28.6%) patients. Acute kidney injury (AKI) was present at diagnosis in 20/65 (30.8%), while 73/233 (31.3%) patients with aPL-N developed chronic kidney disease (CKD)/end-stage kidney disease (ESKD) at follow-up. aPL-N was associated with an increased risk of CKD/ESKD [OR 6.89 (95% CI 2.42–19.58)] and AKI [OR 2.97 (95% CI 1–4-6.29)]. Arterial hypertension and positivity for lupus anticoagulant, anti-cardiolipin antibodies and anti-β2 glycoprotein I antibodies were associated with an increased risk of developing aPL-N [OR 3.7 (95% CI 1.9–7.23), OR 4.01 (95% CI 1.88–8.53), OR 2.35 (95% CI 1.31–4.21) and OR 19.2 (95% CI 2.91–125.75), respectively].

**Conclusion:**

aPL-N is associated with poor renal outcomes. High blood pressure and aPL positivity have been identified as predictors of adverse renal outcomes. This up-to-date knowledge on renal outcomes and predictors of renal outcomes in aPL-N enables a personalized follow-up and therapeutic approach.

## Graphical Abstract

**Graphical Abstract:**
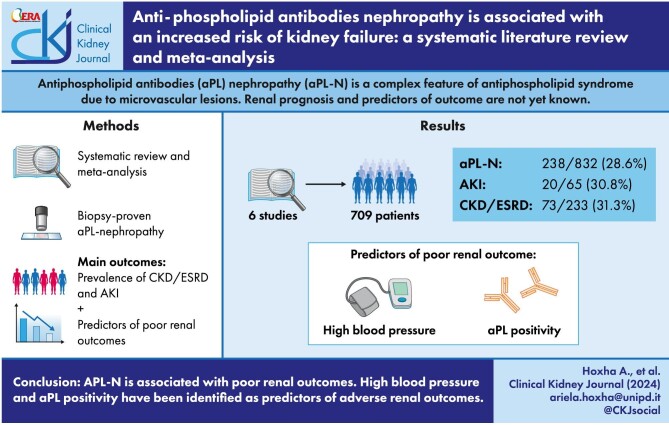

KEY LEARNING POINTS **What was known:**

- The clinical spectrum of anti-phospholipid antibodies nephropathy (aPL-N) is broad, encompassing acute and chronic microvascular changes resulting from different mechanisms, both vascular and non-vascular.
- The prevalence of aPL-N in patients with anti-phospholipid syndrome is not well-defined, with a rating of 6% and 34% based on data from systemic lupus erythematosus studies.

**This study adds:**

- APL-N was associated with an approximately 7-fold and 3-fold increased risk of chronic kidney disease/end-stage kidney disease and acute kidney injury, respectively.
- Arterial hypertension and specific aPL antibodies were identified as predictors of poor renal outcomes.

**Potential impact:**

- Increased awareness of the renal implications of aPL-N among healthcare providers can lead to better surveillance, timely intervention and improved patient outcomes.
- The identified predictors can aid in risk stratification and individualized management of patients with aPL-N, facilitating a proactive approach to renal care in this population.

## INTRODUCTION

Anti-phospholipid syndrome (APS) is a thrombo-inflammatory disorder characterized by vascular thrombosis and pregnancy morbidity in the presence of persistent circulating anti-phospholipid antibodies (aPL) [1].

APS is a challenging disease affecting any organ or tissue, potentially giving rise to a high rate of damage accrual and life-threatening manifestations [2–4].

The entire renal vasculature can be affected by APS, ranging from thrombosis of renal arteries and veins to microvascular injuries involving glomeruli and terminal portions of the arterial vasculature and arterioles [5]. The updated Sapporo consensus criteria classification codified under the term aPL nephropathy (aPL-N) is a broad category of acute and chronic microvascular injuries that may occur due to various vascular and non-vascular mechanisms [2]. The recent American College of Rheumatology (ACR)/European League Against Rheumatism (EULAR) 2023 APS classification criteria included aPL-N in the microvascular domain [6].

Currently the true prevalence of aPL-N is unknown; data, mainly from studies on systemic lupus erythematosus (SLE), report a prevalence ranging from 6 to 34% [7, 8]. Given the risk of haemorrhagic complications due to the possible presence of thrombocytopaenia and concomitant anticoagulant therapy, renal biopsies are considered a high-risk procedure and are performed very rarely. Silvariño et al. [8] reported a significant delay in kidney biopsy in SLE patients who were aPL positive compared with those who were aPL negative (134 ± 60.6 versus 42.6 ± 60.1 months; *P* < .0001).

This means that renal involvement in patients with APS could be a severe complication. Recently, we found that 35% of primary APS (PAPS) patients developed permanent renal impairment and 17.9% required kidney transplantation [4]. Of these, 3 (60.0%) developed renal failure by 7, 10 and 25 years after transplantation, despite anti-thrombotic therapy. None of them had macrovascular involvement.

We performed a systematic literature review to evaluate the prevalence of aPL-N and its clinical, laboratory and histopathologic features. Furthermore, we conducted a meta-analysis to assess the risk of kidney failure in aPL-N compared with those without aPL-N and to determine the clinical and laboratory manifestations suggestive of predictive renal involvement due to aPL.

## MATERIALS AND METHODS

### Literature search

A systematic review of the literature regarding clinical studies of aPL-N was performed according to the Preferred Reporting Items for Systematic Reviews and Meta-Analyses (PRISMA) checklist protocol; the PRISMA checklist is provided in Supplementary Table 1 [9]. Two investigators (H.A. and L.M.) independently performed a detailed search of the PubMed, Scopus, Cochrane Library and Embase databases for original articles, followed by a manual search of pertinent reference lists from the relevant articles retrieved. The period examined was between February 2006 (when the updated classification criteria of APS were published) and January 2024. The search strategy combined free-text search exploring medical subject headings (MeSH/EMTREE) terms to identify relevant published articles: [‘antiphospholipid syndrome’ OR ‘lupus anticoagulant’ OR ‘anti-cardiolipin antibodies’ OR ‘anti-β2 glycoprotein I’] AND [‘nephropathy’ OR ‘ESKD’ OR ‘renal impairment’ OR ‘kidney transplant’. Articles written in languages other than English were excluded.

### Study selection

Selection criteria were determined before data collection. Studies were considered eligible if they met the following criteria: biopsy-proven renal involvement in patients with aPL positivity with or without an APS diagnosis and observational and interventional studies reporting on clinical, laboratory and histopathology data in aPL-N. Reviews, editorials, case reports, case series and clinical, laboratory and histopathology articles of lupus nephritis, as well as articles with poorly or non-documented follow-ups, were excluded. All discrepancies were resolved by consensus between the two principal investigators. A third external reviewer (S.P.) made the final decision in case of discrepancy.

### Data extraction

According to PRISMA guidelines [9], we excluded non-eligible publications based on title and abstract after deleting duplicates. After reviewing the full-text articles, nine articles were included in the systematic literature review [8, 10–17] and six were included in the meta-analysis [8, 10, 12–15]. We retrieved the full text of the selected studies and data were extracted in an ad hoc data extraction form. In case of incomplete or unextractable data, we contacted the corresponding author.

The two investigators simultaneously and independently extracted data. For each selected study, the information recorded included study design, study population, sample size and the following clinical and laboratory features: thrombosis, pregnancy morbidity, acute kidney failure, chronic kidney disease (CKD), hypertension, nephrotic syndrome, haematuria, proteinuria, anti-cardiolipin antibodies (aCL) and anti-β2 glycoprotein I antibodies (anti-β2GPI), lupus anticoagulant (LAC), serum creatine, systolic blood pressure and treatment. Histopathological data were also recorded. Following the ACR/EULAR 2023 APS classification criteria, aPL-N is defined as acute aPL-N characterized by thrombotic microangiopathy lesions such as fibrin thrombi in arterioles or glomeruli without inflammatory cells or immune complex while chronic aPL-N is characterized by chronic/organized arterial or arteriolar microthrombi with or without recanalization, fibrous and fibrocellular arterial or arteriolar occlusions, focal cortical atrophy with or without thyroidization, fibrous intimal hyperplasia (FIH) and chronic/organized glomerular thrombi [18]. Furthermore, the World Health Organization (WHO) classification of lupus glomerulonephritis was recorded [19].

### Quality assessment

The Newcastle–Ottawa scale [20] for cohort studies was used to evaluate the risk of bias for retrospective studies assessing the following items: (1) selection of patients: low if the exposure cohort was very representative of APS patients, the non-exposure cohort was selected from the same community as the exposure cohort, ascertainment of exposure for cases and controls and demonstration that the outcome of interest was not present at start of the study; (2) comparability: low if the cohort studies were comparable; and (3) exposure: low if there was an independent blind assessment and there was an adequate extended/complete follow-up for outcomes to occur. The studies that scored at least seven stars were considered to have high methodological quality, while those with less than seven were considered to have moderate methodological quality.

### Statistical analysis

Statistical analyses were conducted using Stata version 17 (StataCorp, College Station, TX, USA). Data are shown as numbers (percentages). The chi-squared and Fisher's exact tests were performed to evaluate the association between aPL-N and clinical and laboratory features.

The meta-analysis used Review Manager (RevMan) version 5.4 (Cochrane Collaboration, London, UK). Meta-analysis was performed after assessing the homogeneity of designs, populations and outcomes and if at least two studies independently reported a contingency table for the same predictor and outcome. The primary analysis evaluates aPL-N risk to determine CKD/ESKD and acute kidney injury (AKI). We used the odds ratio (OR) as the measure of association in this meta-analysis. Pooled risk estimates were obtained using a random effects model with restricted maximum likelihood (REML) estimations to calculate 95% confidence intervals (CIs). Between-study heterogeneity was calculated using I^2^ (0–100%). According to the Cochrane Handbook [21], an I^2^ ≥75% represents considerable heterogeneity, thus pooled results were reported if I^2^ was <75%. Publication bias was examined using a funnel plot and Egger's regression test [22]. Separate a priori subgroup analyses were planned to evaluate the effect of different clinical and laboratory features in aPL-N. It was not possible to pool analysis for PAPS and secondary APS because only one study enrolled PAPS patients who fulfilled the inclusion criteria for the meta-analysis.

## RESULTS

### Study selection

**Figure 1:**
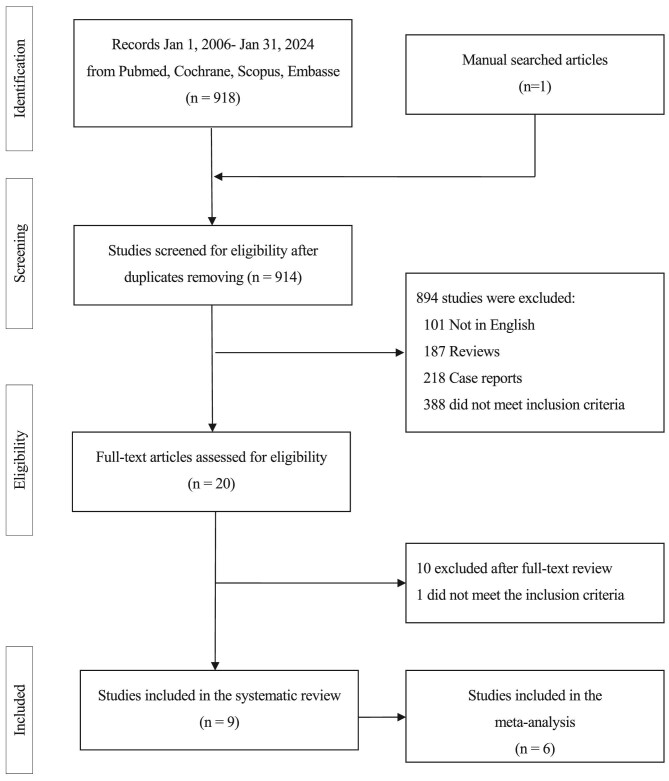
Flow diagram overview of study selection.

After removing duplicates, the search yielded 914 records. Fig. 1 presents the flow diagram of study selection. Nine studies were included in the systematic literature review [8, 10–17] and six were included in the meta-analysis [8, 10, 12–15]. Three studies were not included in the meta-analysis: Tektonidou et al. [11] and Rousselin et al. [16] did not report a contingency table for the same predictor and outcome, and in the study by Sciascia et al. [17], the data on predictor and outcome were not extractable, even after contacting the corresponding author. Table 1 summarizes the studies included in the systematic review of the literature. A total of 899 patients were included in the systematic literature review; 297 (33.0%) fulfilled the updated Sapporo classification criteria for APS [2].

**Table 1:** Overview of clinical studies of aPL-N.

Author, year	Study design	Participants, *n*	Female, *n* (%)	Age (years), mean ± SD	Population	Sydney APS criteria	Biopsy-proven aPL-N, *n* (%)	CKD/ESRD, *n* (%)	Follow-up (years), mean ± SD	Deaths, *n* (%)
Cheunsuchon et al., 2007 [10]^b^	Retro	150	114 (76)	22.9 ± 11.4	SLE	24 (16)	51 (34)	17 (33)	NR	3 (5.9)
Tektonidou et al., 2008 [11]	Retro	37	26 (70.3)	37 ± 4.9	PAPS, CAPS, SLE-APS	37 (100)	37 (100)	9 (27.3)	6.1 ± 1.4	4 (10.8)
Miranda et al., 2009 [12]^b^	Retro	162	144 (88.9)	27.6 ± 8.1	SLE	NR	17 (10.4)	5 (29.4)	7.0 ± 4.4	9 (52.9)
Sinico et al., 2010 [13]^b^	Retro	160^c^	140 (87.5)	35 ± 12	PAPS	160 (100)	10 (6.3)	3 (30)	8.3 ± 7.1	0 (0)
Silvariño et al., 2011 [8]^b^	Retro	77	70 (88.6)	33.3 ± 11.6	SLE	NR	9 (11.4)	3 (33.3)	6.1 ± 4.3	0 (0)
Erre et al., 2014 [14]^b^	Retro	48	41 (85.4)	36 (14–66)^a^	LN	7 (14.6)	12 (25)	2 (16.6)	6 (2–13)^a^	1 (8.3)
Gerhardsson et al., 2015 [15]^b^	Retro	112	89 (79.5)	38 (18–84)^a^	SLE	13 (11.6)	16 (14.2)	6 (37.5)	5.9 years	NR
Rousselin et al., 2022 [16]	Retro	30^c^	19 (63.3)	40 (34–52)^a^	aPL positive	15 (50)	30 (100)	6 (20)	6.2 (1.8–9.1)^a^	1 (3.3)
Sciascia et al., 2023 [17]	Retro	123^c^	101 (82.1)	NR	aPL positive	23 (41.1)	56 (45.5)	23 (41.1)	12 months from biopsy	NR

SD: standard deviation; CAPS: catastrophic anti-phospholipid syndrome; Retro: retrospective study; NR: not reported.

a Median (interquartile range).

b Included in the meta-analysis.

c Multicentre studies.

### Assessment of quality score and publication bias

**Figure 2:**
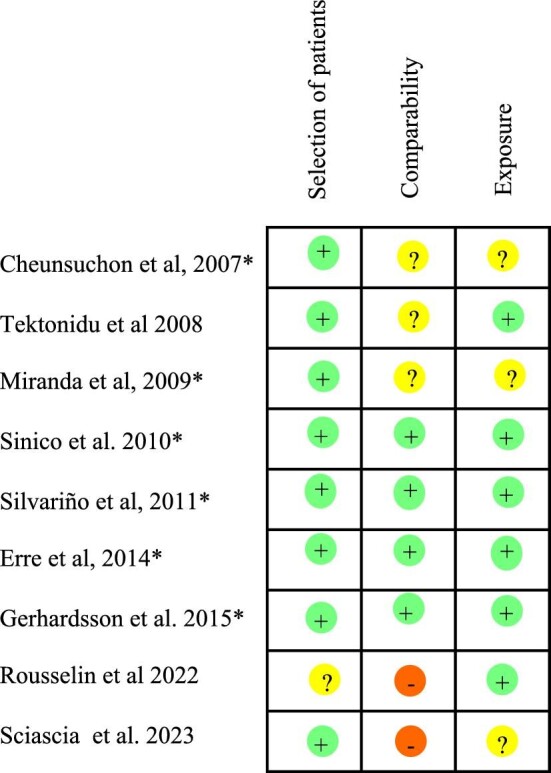
Quality assessment of the studies included in the systematic literature review. *Studies included in the meta-analysis.

The risks of bias are reported in Fig. 2. The quality score was assessed with the Newcastle–Ottawa scale for cohort studies [20], which assigned a high-quality rating to four studies [8, 13–15] and a moderate-quality rating to five studies [10–12, 16, 17]. Furthermore, as shown in Supplementary Fig. 1, a visual examination of the funnel plots for the primary analysis showed no asymmetry and the Egger's test (*P* = .6) did not reveal any statistical evidence for publication bias.

### Clinical and laboratory characteristics of aPL-N

The patients’ characteristics at diagnosis are reported in Table 2. Biopsy-proven aPL-N was found in 238/832 patients (28.6%). AKI was present at diagnosis in 20/65 (30.8%), while 73/233 (31.3%) patients with aPL-N developed CKD/ESKD at follow-up. Both AKI and CKD/ESKD were significantly more common in aPL-N patients than those without aPL-N [OR 2.7 (95% CI 1.3–5.6), *P* = .007 and OR 3.8 (95% CI 2.6–5.8), *P* < .0001, respectively]. Arterial hypertension and mild proteinuria (<2.5 g/24 h) were the clinical manifestations that presented most often at diagnosis, at 73% and 66.7%, respectively. Arterial hypertension and proteinuria were significantly more prevalent in patients with aPL-N than those without [OR 2.8 (95% CI 1.8–4.5), *P* < .0001 and OR 11.9 (95% CI 6.1–22.5), *P* < .0001, respectively]. Furthermore, nephrotic syndrome was significantly more common in patients with aPL-N compared with those without aPL-N [OR 1.9 (95% CI 1.2–3.0), *P* = .008]. Patients with a history of thrombosis had an almost 6-fold higher risk of developing aPL-N [OR 5.6 (95% CI 3.4–9.3), *P* < .0001]. Pregnancy morbidity, encountered in 11.1% of aPL-N, was not associated with aPL-N. aCL antibodies were found in 79.4% of aPL-N patients. aCL antibodies, as well as anti-β2GPI and LAC, showed a significantly higher prevalence in aPL-N [OR 5.5 (95% CI 3.34–9.5), *P* < .0001, OR 3.3 (95% CI 1.7–6.4), *P* = .0004 and OR 5.7 (95% CI 2.1–8.5), *P* < .0001, respectively]. Triple aPL positivity was recorded in 39.8% of patients. The patients with triple aPL positivity had a 6-fold higher risk of developing aPL-N [OR 6.5 (95% CI 3.4–12.4), *P* < .0001].

**Table 2:** Clinical and laboratory features at diagnosis of patients with aPL-N.

Features	aPL-N (*n* = 238), *n* (%)	No aPL-N (*n* = 661), *n* (%)	*P*-value
CKD/ESKD	73 (31.3)	52 (10.6)	<.0001
AKI	20 (30.8)	19 (13.9)	.007
Arterial hypertension	92 (73.0)	155 (48.7)	<.0001
Haematuria	42 (51.9)	28 (68.3)	NS
Proteinuria	82 (66.7)	15 (14.4)	<.0001
Nephrotic syndrome	46 (38.7)	71 (24.9)	.008
Thrombosis	82 (44.6)	26 (12.6)	<0.0001
Pregnancy morbidity	9 (11.1)	6 (14.6)	NS
LAC	72 (66.1)	44 (25.6)	<.0001
aCL	100 (79.4)	102 (40.9)	<.0001
anti-β2GPI	37 (43.5)	19 (19)	.0004
Triple aPL	39 (39.8)	15 (9.2)	<.0001

### Histopathological findings of aPL-N

Table 3 highlights the histopathological characteristics of aPL-N at diagnosis. Acute lesions, defined as thrombotic microangiopathy, were present in 102/210 patients (48.6%). The most frequent chronic lesion was FIH, presenting in 138/208 patients (66.3%). Interestingly, 69/111 biopsies (62.2%) showed the co-existence of membranous glomerulonephritis.

**Table 3:** Histopathological characteristics of aPL-N at diagnosis.

Characteristics	aPL-N (*N* = 238), *n*	%
Acute lesions		
TMA (*n* = 210)	102	48.6
Chronic lesions		
Fibrous intimal hyperplasia (*n* = 208)	138	66.3
Focal cortical atrophy with/without thyroidization (*n* = 154)	66	42.9
Organized arterial or arteriolar microthrombi (*n* = 183)	37	20.2
Fibrous and fibrocellular arterial or arteriolar occlusions (*n* = 118)	61	51.7
Glomerulonephritis WHO classification		
Class I (*n* = 111)	0	0
Class II (*n* = 111)	3	2.7
Class III (*n* = 111)	5	4.5
Class IV (*n* = 111)	69	62.2
Class V (*n* = 111)	18	16.2
Class VI (*n* = 111)	2	1.8

### Meta-analysis

#### The risk of developing renal insufficiency in aPL-nephropathy

**Figure 3:**
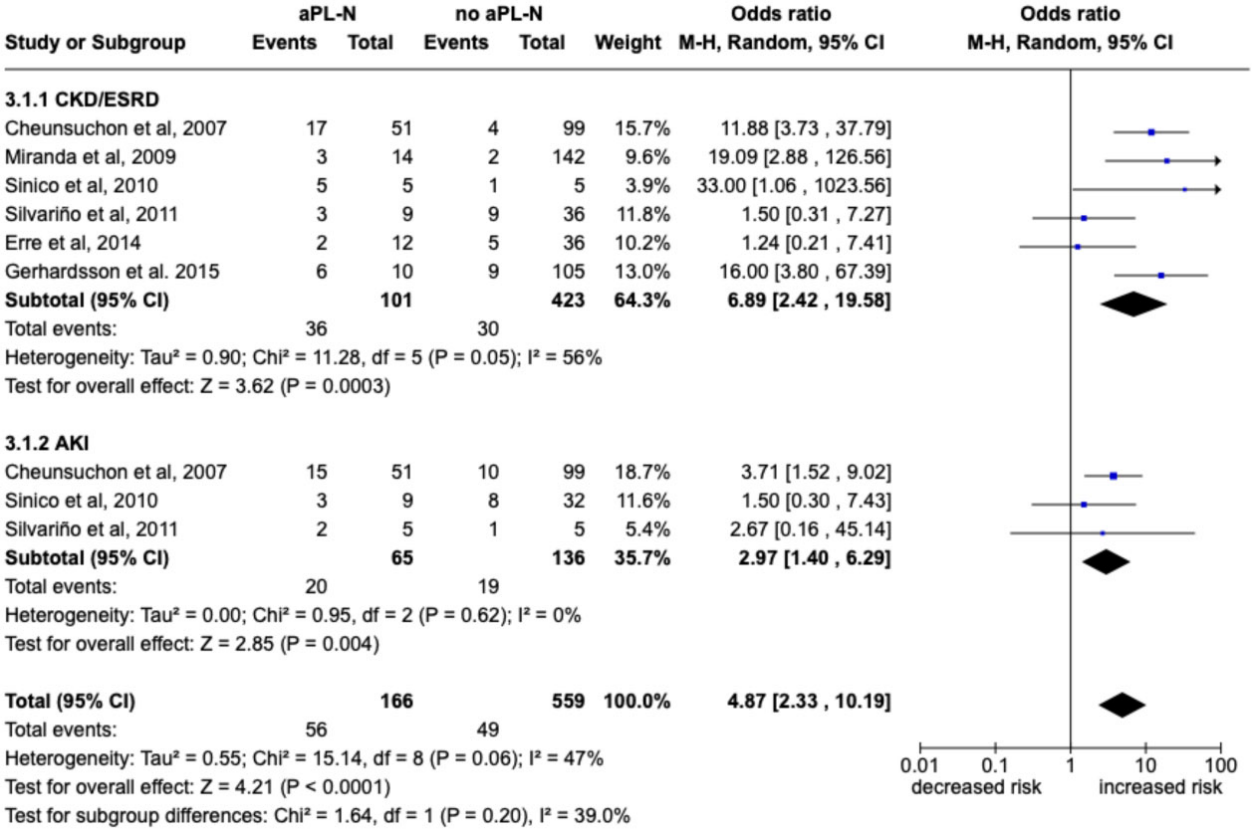
Forest plot for the risk of kidney failure in aPL-N. Pooled ORs for CKD/ESRD and AKI are reported with 95% CI, number of pooled studies, number of observations, heterogeneity and test of overall effect.

Overall, 709 patients were included in the meta-analysis and 207 (29.2%) fulfilled the updated Sapporo classification criteria [4]. As shown in Fig. 3, aPL-N was associated with an increased risk of CKD/ESKD [OR 6.89 (95% CI 2.42–19.58)] and AKI [OR 2.97 (95% CI 1.4–6.29)]. It was not possible to perform a pooled subanalysis according to the diagnosis of PAPS versus SLE, as only one study [13] satisfying the inclusion criteria for the meta-analysis considered PAPS patients.

#### Clinical and laboratory features predictive of aPL-N

**Figure 4:**
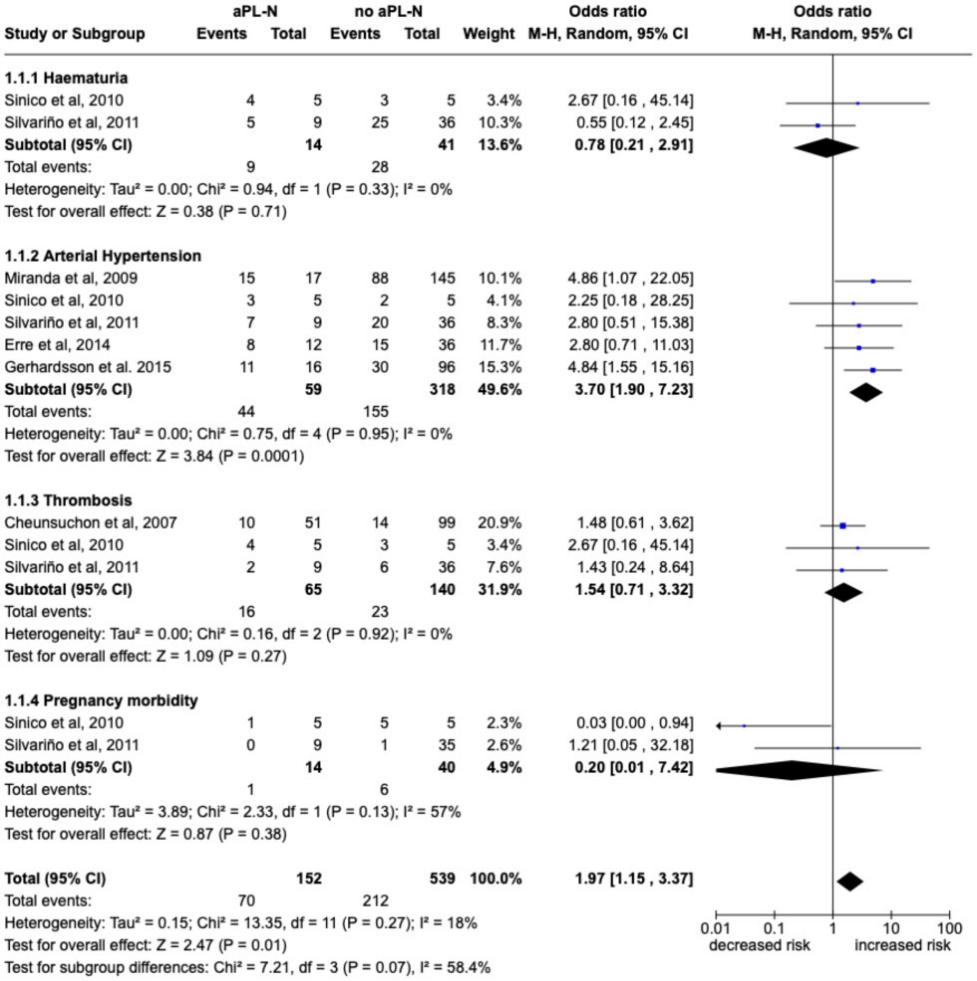
Forest plot for the risk of aPL-N according to clinical features predictors. Pooled ORs for each predictor are reported with 95% CI, number of pooled studies, number of observations, heterogeneity and test of overall effect.

Haematuria (*n* = 2), arterial hypertension (*n* = 5), previous thrombosis (*n* = 3), proteinuria (*n* = 2) and nephrotic syndrome (*n* = 4) were studied as potential clinical predictors of aPL-N [8, 10, 12–15]. As shown in Fig. 4, patients with arterial hypertension at diagnosis had a significantly increased risk of developing aPL-N [OR 3.7 (95% CI 1.9–7.23)]. At the same time, haematuria, a history of previous thrombosis and pregnancy morbidity were not associated with an increased risk of aPL-N. Pooled analysis was not possible for proteinuria and nephrotic syndrome due to the high heterogeneity of I^2^ = 81% and I^2^ = 84%, respectively.

**Figure 5:**
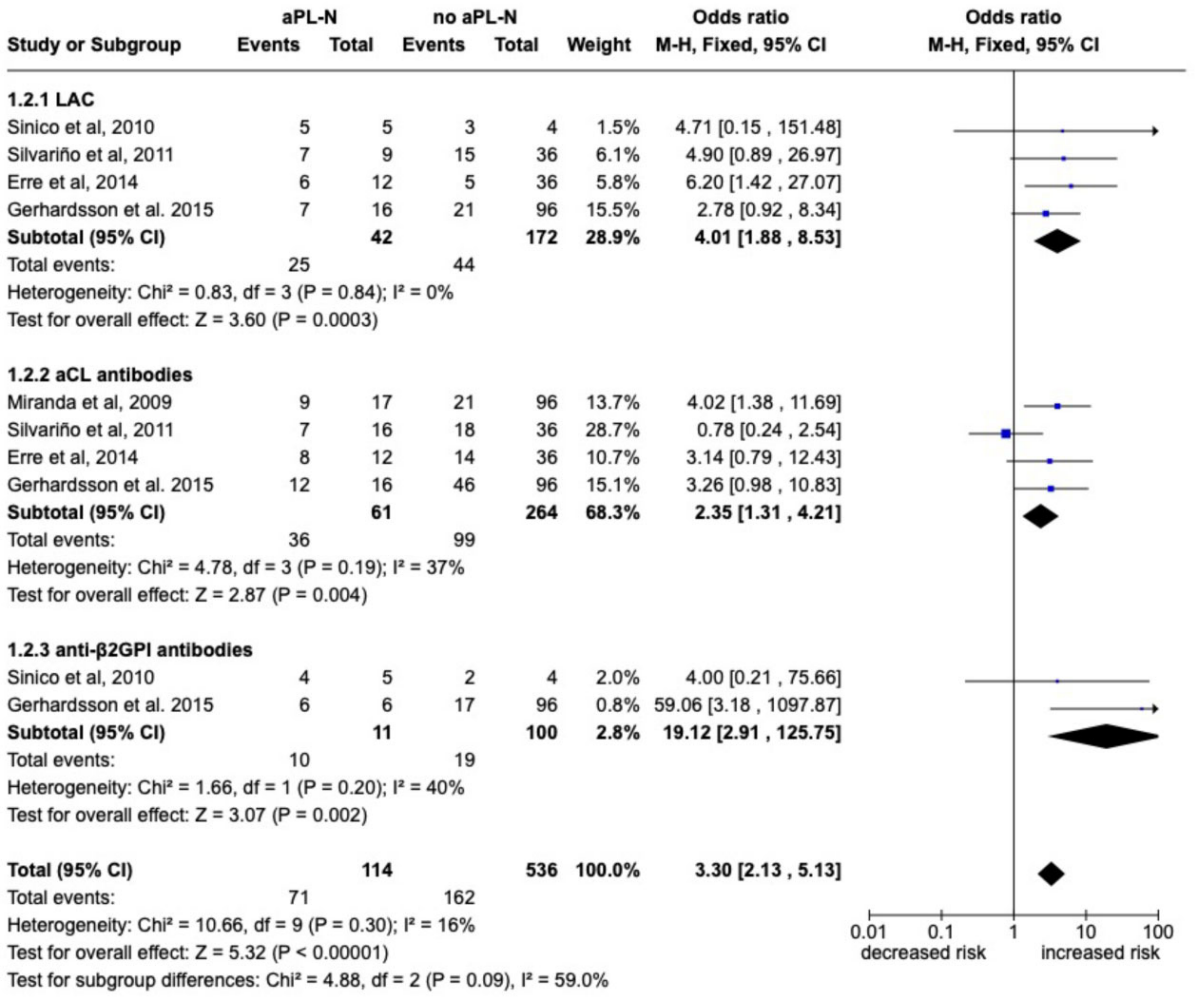
Forest plot for the risk of aPL-N according to laboratory features predictors. Pooled ORs for each predictor are reported with 95% CI, number of pooled studies, number of observations, heterogeneity and test of overall effect.

LAC (*n* = 4), aCL (*n* = 4) and anti-β2GPI (*n* = 2) antibodies were studied as potential laboratory predictors of aPL-N [8, 12–15]. As reported in Fig. 5, patients with LAC, aCL and anti-β2GPI antibodies had a significantly higher risk of developing aPL-N [OR 4.01 (95% CI 1.88–8.53), OR 2.35 (95% CI 1.31–4.21) and OR 19.2 (95% CI 2.91–125.75), respectively]. Pooled analysis regarding triple aPL positivity was not possible because only one study [15] reported the association between aPL-N and triple aPL positivity.

## DISCUSSION

This systematic literature review investigated the prevalence and risk of kidney failure in aPL-N and identified clinical and laboratory manifestations suggestive of renal involvement due to aPL. Overall, a prevalence of 28.6% for aPL-N was seen among patients with aPL positivity who fulfilled APS classification criteria or not. Pooled estimated analysis showed that patients with aPL-N have a 7-fold and 3-fold higher risk of CKD/ESKD and AKI, respectively, compared with those without aPL-N. Previously, data showed that aPL antibodies in patients with lupus nephritis were associated with poor renal outcomes during follow-up [22]. However, although two studies found evidence of aPL-N in 32% and 23.2% of SLE patients, they did not demonstrate an association between aPL-N and renal outcome [23, 24]. Tektonidou et al. [25] showed that arterial hypertension and elevated serum creatinine levels at diagnosis were associated with poor renal outcome. Indeed, the patients with aPL-N compared with those without aPL-N had higher rates of hypertension and elevated serum creatinine levels at diagnosis [OR 6.7 (95% CI 2.4–18.7) and OR 4.0 (95% CI 1.4–11.2), respectively]. A retrospective multicentre study recently reported ESKD in 20% of isolated aPL-N, with an ESKD-free survival rate of 80% at 5 years and 72.7% at 10 years [16]. Once again, we found permanent renal impairment in 35% of the PAPS patients, and almost 18% developed ESKD. These controversial data reflect the study designs, populations enrolled and sample sizes. However, this systematic review of the literature and meta-analysis demonstrated for the first time that aPL-N is associated with an increased risk of kidney failure, as one-third of the patients with aPL-N reported kidney failure at diagnosis. This emphasizes the need for early diagnosis to give these patients the possibility of adequate treatment to prevent permanent kidney damage.

Arterial hypertension, non-nephrotic proteinuria and haematuria were the most common renal manifestations reported in previous studies [5, 24]. The pooled analysis found that arterial hypertension was associated with a 4-fold higher risk of aPL-N. Haematuria did not result in an increased risk of aPL-N. Regarding proteinuria and nephrotic syndrome, a pooled analysis was not possible due to the higher heterogeneity of the studies. However, together with arterial hypertension, non-nephrotic proteinuria was found to have a significantly higher prevalence at diagnosis of aPL-N compared with patients without aPL-N. Also, nephrotic syndrome was significantly more prevalent in aPL-N than in patients without aPL-N (38.7% versus 24.9%), possibly reflecting the study population with a prevalence of SLE-APS patients. A history of previous thrombosis was found in 44.6% of aPL-N patients. Even though the pooled analysis showed a tendency towards an increased risk of aPL-N in thrombotic APS patients, it failed to reach statistical significance, possibly due to the sample size. However, when we considered the studies overall, a history of thrombosis was associated with a 6-fold higher risk of developing aPL-N. Also, a recent multicentre study reported that thrombosis and triple-positive aPL have the worst renal prognosis [17]. Indeed, by performing cluster analysis, they identified the so-called thrombotic microangiopathy (TMA) cluster characterized by a higher frequency of thrombotic APS and triple aPL positivity (91.3% and 65.2%, respectively) and associated with poor renal outcome.

In contrast, the ‘subendothelial oedema cluster’, characterized solely by SLE patients and a few cases of associated APS had a favourable renal outcome [17]. Pooled analysis regarding triple aPL positivity was not possible, as only one study reported data in this regard. However, triple aPL positivity was significantly associated with aPL-N in the univariate analysis. Also, Gerhardsson et al. [15] found a significantly higher prevalence of triple aPL positivity in patients with aPL-N versus patients without aPL-N (37.5% versus 12.5%; *P* = .02).

Regarding laboratory features, the pooled analysis demonstrated that both aCL and anti-β2GPI antibodies were associated with an increased risk of aPL-N. Pooled analysis regarding LAC showed an increased risk of aPL-N compared with those without aPL-N. Controversial data are reported in the literature. If, on the one hand, the presence of LAC associated with aPL-N is confirmed throughout the literature, the presence of aCL presents conflicting data. A single-centre Spanish study of the renal biopsies of 79 SLE patients reported aCL antibodies associated with aPL-N compared with those without aPL-N (77.8% versus 28.1%; *P* = .002) [8]. In contrast, a Greek study, again on SLE patients, did not find an association between aCL antibodies and aPL-N [24].

Regarding histopathological findings, TMA was present in almost half of aPL-N biopsies and FIH was present in two-thirds of aPL-N biopsies, representing the most prevalent chronic lesions. We could not evaluate potential associations between histological features of aPL-N and renal outcome. However, a multicentre French study found the extent of acute tubular necrosis associated with an estimated glomerular filtration rate decline at 12 months of follow-up [16]. Moreover, an international multicentre study reported that the cluster ‘TMA’ resulted in a higher rate of non-response than those of ‘hyperplastic vasculopathy’ (47.8% versus 36.4%) [17].

Our study has several limitations, which intrinsically depend on the meta-analysis and the quality of the included studies. First, due to the relatively small number and retrospective design of most of the studies in this meta-analysis, the results of this study are subject to random error. Second, the sample size of studies can also influence the fidelity of the results, especially concerning subgroup analysis of clinical and laboratory characteristics. Third, clinically heterogeneous data due to enrolment (PAPS, SLE-aPL positive, aPL positive) may have led to different therapeutic approaches that impacted the renal outcome, opening the strong possibility of bias and making the quality of most studies in this meta-analysis low. Unfortunately, it was not possible to perform pooled analysis on the treatment because it was reported inconsistently. However, in favour of the results of our study, one-third of patients already had CKD/ESKD at the diagnosis of aPL-N. Lastly, we could not perform pooled analysis on triple aPL positivity and serum creatinine levels, dsDNA, and C3, C4, which could be markers of renal outcomes, due to inconsistency across the studies.

In conclusion, this meta-analysis summarizes the current evidence on the impact of aPL-N on renal outcome and available predictors of renal outcome. Pooled analysis showed that aPL-N is associated with poor renal outcomes. High blood pressure and aPL positivity have been identified as predictors of adverse renal outcomes. Methodologically rigorous multicentre studies on aPL-N in PAPS patients are urgently needed. Further research agendas should include population-based studies to determine the prevalence and incidence of APS nephropathy, characterize the clinical presentation of APS nephropathy and identify factors that influence the progression and outcome of aPL-N. Also, longitudinal studies to evaluate long-term kidney function outcomes are needed.

Another critical point to further investigate is the impact of coexistent autoimmune diseases, like SLE, on kidney prognosis and the interaction between aPL- N and hypertension. Further, standardized diagnostic criteria and biomarkers for early detection should be developed. Technological advancements in digital pathology, machine learning and omics might be employed to help understand and diagnose aPL-N. Meanwhile, the presented results provide clinicians with updated knowledge on renal outcomes and predictors of renal outcomes in aPL-N, enabling a personalized follow-up and therapeutic approach.

## Supplementary Material

sfae302_Supplemental_Files

## Data Availability

All data generated or analysed during this study are included in the article and its respective cited articles.
