# Supplementary figures and images for: Anti-phospholipid antibodies nephropathy is associated with an increased risk of kidney failure: a systematic literature review and meta-analysis
Source: Clin Kidney J. 2024 Oct 7;17(10):sfae302. doi: 10.1093/ckj/sfae302 (PMC11500446; doi:10.1093/ckj/sfae302)

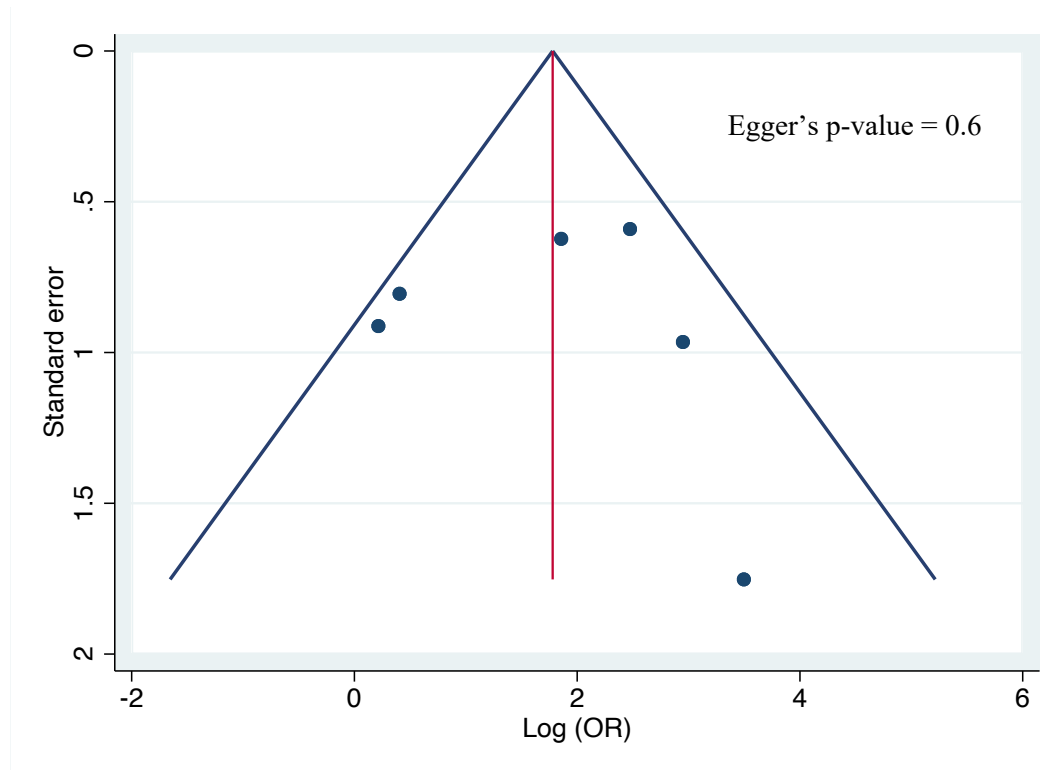

**Suppl. Figure 1** Hoxha et al, 2024

Supplement: sfae302_Supplemental_Files [file sfae302_supplemental_files.zip › Hoxha_ Suppl_Figure_1_ to_go.pdf]
